# Psychosocial challenges affecting the quality of life in adults with epilepsy and their carers in Africa: A review of published evidence between 1994 and 2014

**DOI:** 10.4102/phcfm.v9i1.1275

**Published:** 2017-03-30

**Authors:** Mpoe J. Keikelame, Tamzyn Suliaman, Marleen Hendriksz, Leslie Swartz

**Affiliations:** 1 Primary Health Care Directorate, Groote Schuur Hospital, South Africa; 2 Department of Psychology, Stellenbosch University, South Africa; 3 Health Sciences Faculty, University of Cape Town, South Africa

## Abstract

**Background:**

Little attention has been paid to the psychosocial challenges of adult patients with epilepsy and their carers in Africa in published studies conducted between 1994 and 2014 – yet these psychosocial challenges have been reported to have a major impact on the quality of life (QOL) of people living with the illness and those who care for them.

**Aim:**

This review aimed to examine the literature on published studies conducted in Africa between 1994 and 2014 that examined psychosocial challenges of adult people with epilepsy and their carers. The aim was to examine the kind of psychosocial challenges from published evidence and to identify gaps in current knowledge.

**Methods:**

MEDLINE, PubMed, ERIC, Web of Science, Scopus, Cochrane Library, Africa Wide, PsycINFO, EMBASE, PASCAL, SABINET and Google Scholar databases and hand searches of *Epilepsy & Behavior, Epilepsia, South African Medical Journal, African Journal of Disability* and *African Journal of Primary Health Care and Family Medicine* were reviewed.

**Results:**

Very few studies in Africa have examined psychosocial challenges affecting the adult patients with epilepsy and their carers. This review reported the existence of evidence of such challenges and the insights into the psychosocial and economic factors that underpin them.

There is evidence that where these have been addressed, there were valuable insights on the types of psychosocial, socio-cultural and socio-economic challenges. Collaborative empowering interventions are needed to enable the attainment of good QOL for those affected.

**Conclusion:**

Research on psychosocial challenges of adult patients with epilepsy and their carers from different sectors of healthcare to inform the design of appropriate models is needed.

## Introduction

Literature shows that people with epilepsy (PWE) experience psychosocial difficulties, which have a huge impact on their quality of life (QOL).^1^ QOL is experienced subjectively and includes the positive and negative dimensions that are embedded in a cultural, social and environmental context. It is also characterised by six domains: physical, psychological, level of independence, social relationships, environment and spirituality/personal beliefs.^2^ Recognising the impact of epilepsy on the QOL of PWE and their families, the WHO International League Against Epilepsy (ILAE), the International Bureau of Epilepsy and global initiatives such as the Out of the Shadows Campaign^3^ were initiated. Currently, epilepsy accounts for about 0.7% of the global burden of disease with about 0.261% of the total worldwide burden being in Africa.^4^ The World Health Assembly’s resolution on the global burden of epilepsy has recently approved that all countries should address the health, social and public knowledge factors impacting on epilepsy through co-ordinated actions.^5^ This review aimed to gain insight into existing knowledge. The evidence from reviewed studies on psychosocial, cultural and socio-economic–related challenges indicated that PWE and their carers struggle in Africa with identifiable gaps in current knowledge, hence an avenue for future research. The following were the review questions:

- What are the psychosocial challenges that adult PWE and their carers struggle with in order to attain a good QOL?
- What are the gaps in current knowledge?

## Methods

An electronic search was carried out using MEDLINE, PubMed, ERIC, Web of Science, Scopus, Cochrane Library, Africa Wide, PsycINFO, EMBASE and PASCAL, SABINET and Google Scholar to avoid missing other important articles.^6^ In addition, hand searches were conducted of published brief communications, letters to the editor, journals such as *Epilepsy & Behavior, Epilepsia* (the ILAE journal), *Gray Matters from Epilepsia*, African journals such as the *South African Medical Journal, African Journal of Disability* and the *African Journal of Primary Health Care and Family Medicine*. Retrieved articles written in English were reviewed using the preview, question, read and summarise method and were extracted into a matrix for analysis, synthesis and interpretation.^7^

## Review findings

### Epilepsy terminology and meanings

The importance of understanding lay cultural explanations of epilepsy and the implications for the lack thereof is well explained by Fadiman.^8^ This is quite important because explanatory models of illness held by lay people and those held by western trained healthcare professionals may differ^9^ and this may have an impact on the treatment and care of patients in general. Taking an historical approach, Jilek-Aall^10^ described terminologies that have been used to refer to epilepsy and types of seizures. There was evidence of a wide range of lay terms that are used to explain epilepsy and seizures, which differed in the same country and between countries – and had different interpretations and meanings, which can lead to misunderstanding of the illness and its treatment and care. Further research is needed in this area.

### Issues of people with epilepsy

#### Understanding medical language and epilepsy information

Medical language can pose difficulties for PWE and carers in their understanding of epilepsy information. In Zimbabwe, Saburi^11^ found that the word ‘temporal’ as in ‘temporal lobe epilepsy’ was understood as ‘temporary seizures’ by one parent. Similarly, there was also evidence of misinterpretation of types of seizures in a South African study by McQueen and Swartz.^12^ They found that the suffix ‘mal’ in petit-mal and grand-mal seizures was interpreted as ‘mad’ because the word ‘mal’ in the Afrikaans language means ‘mad’.

It was also of note that lack of access to appropriate and evidence based information can lead to poor understanding of the illness and mistreatment of PWE. Data from the study by Mugumbate and Nyanguru^13^ show a need for pre-testing or evaluating mass media materials that convey information on epilepsy because these can reinforce negative attitudes and inappropriate actions towards PWE. The authors particularly referred to epilepsy information that was conveyed via a video documentary, *Pfari muZimbabwe*, which showed a PWE who was tied to a tree as a form of isolating him from the community. Similarly, Adjei et al.^14^ found that a man who had epilepsy in late adulthood was subjected to neglect and marginalisation in social participation – probably because of a lack of factual information regarding who can get epilepsy and at what age a person can get the illness. These findings show a need for formative research to ensure appropriateness of the content of epilepsy information materials.

#### Employment and driving

Our review findings showed marginalisation of PWE in terms of employment and driving. In South Africa, McQueen and Swartz^12^ found that there were some PWE who were unable to access disability grants (a governmental form of financial support) and were also unable to get employment in protective workshops of the local epilepsy organisation. In another South African study, Segar^15^ found that despite the availability of these grants, PWE have difficulty in accessing them because access thereof depended on compliance and on medical doctors’ decisions to either motivate for grants or not. The author further states that PWE, whose seizures were poorly controlled and who may request a written motivation to access these grants, would either be viewed as beggars for an income or beggars for a free meal ticket, or faking seizures with no proper evaluation of their social circumstances.^15^

In terms of driving, one of the most troublesome issues that were reported by Sunmonu et al.^16^ in Nigeria was that some PWE were of the view that they could ‘double’ the seizure medication dose in order to drive. This finding further highlights issues on which PWE should be counselled and those that need to be considered when designing the content for educational interventions.

#### Stigma

Stigma of PWE has been reported as a global phenomenon.^17,18^ In their study investigating social determinants of felt stigma in Zambia, Atadzhanov et al.^18^ found that felt stigma was because of forced disclosure, beliefs and misperceptions about contagion. The authors further highlighted a need for development of appropriate messages that can decrease misperceptions about contracting epilepsy. The review data further support the idea of the importance of promoting awareness about patients’ health rights because forced disclosure could be a form of violation of such rights.

We found that medical diagnosis of epilepsy can be a potentially stigmatising label for PWE. For example, Allotey and Reidpath^19^ reported that in their study PWE were identified as ‘epileptics’– which may reduce PWE to ‘things’ or ‘bearers of things’. Research is therefore needed to explore the impact of these medical labels on PWE and how these may perpetuate stigma.

#### Physical and sexual abuse

Abuse of women with epilepsy (WWE) has been reported in three reviewed studies done in Zambia by Birbeck et al.^20^ and in Nigeria by Komolafe et al.^21,22^ as one of the key factors that affect the QOL of WWE. On the other hand, Komolafe et al.^22^ found that in cases where some WWE consulted with traditional healers and remained under their care, they were often abandoned by their families and some were sexually abused by some of the healers. There were other factors that were reported in a study in Zambia in 2008^20^ such as lack of just laws to protect WWE from these abusive actions and lack of financial support for divorced WWE, which can lead WWE to abject poverty. In addition, there was also evidence that less attention has been paid to investigating abuse of men with epilepsy.^20^ While advocacy actions for just laws were reported by Birbeck et al.^20^ the findings suggest a need for educational interventions on abuse, recognition thereof and interventions to empower WWE and their carers with skills and support systems to deal with the problem.

#### Transactional sex

This sexual practice has been reported in Uganda by Choudhry et al.^23^ as a sexual practice, which is different from formal commercial sex work and is one of the main sources of the spread of HIV/AIDS in Sub-Saharan Africa. According to these authors, transactional sex involves agreements in exchange of favours such as gifts or money and exist between older women and young males (sugar mommies) and between older men and young girls (sugar daddies) as well as among youth.^23^ In WWE, there was evidence of transactional sex in two reviewed studies conducted in Nigeria by Komolafe et al.^21^ and in Zambia by Birbeck et al.^20^ and has been related to limited financial support, social exclusion, abandonment and marginalisation of WWE. Komolafe et al.^21^ caution that even though this sexual activity may be viewed as different from commercial sex, it may lead to promiscuity and unintended sexual risk in WWE. However, authors reported that WWE denied this view because they reported having other means of income – details of which the authors did not provide. Analysis of the data shows the need to exclude HIV/AIDS-related seizures in older women and men who may report with a history of seizures.

#### Marriage

In our reviewed data, an interesting finding was that marriage difficulties can be perpetuated by significant others such as mothers-in-law. Komolafe et al.^22^ in Nigeria reported on this issue where daughters-in-law who present with epilepsy after marriage are often abandoned and rejected – the main perpetrators being the mothers-in-law. This powerful role of the mother-in-law has been portrayed in a number of African movies and television shows aired on the continent (see, for example, ‘Africa Magic’ http://africamagic.dstv.com/africa-magic-movies). This finding suggests a need for research on how television shows and movies may possibly be used to promote norms that are enabling rather than those that inhibit the health and well-being of WWE. Other marriage-related factors such as sexual dysfunction and polygamous relationships were reported by Birbeck et al.^20^ as factors that pose challenges for WWE – but these were not fully discussed.

### Issues of popular carers

According to Nuhu et al.^24^ caregivers of PWE are often forgotten in epilepsy research. In their study in Nigeria that examined the caregiver burden using the Zarit Burden Interview (ZBI) instrument, there was evidence of higher burden scores among carers who cared for young and unemployed adults who lived in rural areas and whose course of epilepsy was of long duration. Their review findings show that caregivers of PWE face major health and socio-economic challenges, which are because of factors such as polygamous marriages, poor marital relationships, lack of financial support and traditional norms, which restrict caregiving roles to women. On the other hand, Adjei et al.^14^ in Northern Ghana found that a female carer who was economically empowered was believed to have gained *evil wealth* by engaging in witchcraft-related actions, which involved inflicting her child with epilepsy.

### Gaps in research

From our analysis of the reviewed articles, it was evident that there were gaps regarding studies addressing psychosocial and cultural issues affecting adult PWE and their carers.

#### Studies focusing on folk carers

While literature shows that there are different types of traditional healers (THs),^25^ in this reviewed data very few studies highlighted the different types of healers – and categorising all folk carers under one umbrella term such as THs may suggest that they are similar – yet they differ in their training and their approach to treatment and practice.^25^ In addition, these healers have been recommended by the WHO mental health Global Action Programme plan 2013–2020 as a potential resource to reduce the treatment gap in lower- and middle-income countries.^26^ We found only four studies that examined epilepsy among these healers.^27,28,29,30^ Yet there is evidence that THs can play a crucial role in epilepsy treatment and care.^27,28,30^

#### Studies focusing on community-based rehabilitation volunteers

In this review, we found only one cross-sectional study by Otte et al.^31^ that explored community-based rehabilitation volunteers’ (CRVs) familiarity, knowledge and attitudes towards epilepsy in Guinea-Bissau. CRVs were Christians but there was a lack of attention to their Christian values and how these may influence their care of PWE. Other concerns were that despite CRVs’ not believing that epilepsy was a sign of intellectual disability, they suggested that children with epilepsy (CWE) should be educated in special schools – but the reason for this view was not stated. One of the CRVs was of the view that high consumption of protein (specifically eggs) is an effective treatment for epilepsy – it was unclear if this view was held by all participants.

#### Studies focusing on professional carers

There were very few articles that examined different parameters on epilepsy among a variety of healthcare workers (HCWs) in Cameroon in^32,33^ Zambia,^34^ Zimbabwe^35^ and Ghana.^14^ However, these aspects were not fully examined – yet they have been raised as important issues for western trained healthcare professionals because of the concern that they are most often ignored.^36^ For example, in Malawi, Munthali et al.^37^ found that one of the study participants reported being advised by a HCW to stop seizure medication and to use traditional/spiritual treatments – showing a need for research in this area. Another one reported on observations made by Ratsimbazafy et al.^38^ in religious camps in Madagascar and noted that first aid actions for PWE during a seizure attack involved isolation and shackling treatment. The authors were of the opinion that these first aid actions could be influenced by religious beliefs held by HCWs. The finding of these reports shows the need for research to explore these religious factors among HCWs.

Adjei et al.^14^ reported in their study on perceptions of HCWs about their communities’ views on epilepsy. Although this study did not focus on HCWs perceptions per se, perception such as that ‘men who develop epilepsy have worms in their anuses’ were of concern. Although authors thought that this perception could be because of onchocerciasis, which is endemic in Northern Ghana, this finding indicates a need for research especially on how these perceptions may affect the status, relationships and roles of men who have epilepsy.

#### Studies focusing on popular carers and people with epilepsy

Very few studies that examined different parameters of these aspects focused only on popular carers of PWE and WWE. There were also very few qualitative and quantitative studies that we found that examined different parameters of these aspects using different research designs and instruments. Only one qualitative study conducted in Cameroon by Allotey and Reidpath^19^ used a mixed ethnographic method, which included Photo Voice to explore the socio-cultural and environmental issues affecting PWE.

#### Studies using instruments for assessment of psychosocial issues

We found five studies that used different instruments to assess different parameters of these aspects among adult PWE. A study by Nubukpo et al.^39^ used the Goldberg’s Anxiety and Depression scale (GADS); Rafael et al.^40^ used mixed instruments such as the Explanatory Model Interview Catalogue, GADS and Jacoby Stigma scale; Olley and Osinowo^41^ in Nigeria used the Washington Psychosocial Seizure Inventory, ZBI by Nuhu et al.^24^ and Yusuf et al.^42^ used the Hospital Anxiety and Depression Scale – thereby showing a gap in this area. Only one qualitative study using Kleinman’s^9^ Explanatory Model framework was conducted by Mushi et al.^43^ and would be appropriate for exploring these aspects in more detail from the lay and professional carers’ point of view.

#### Studies focusing on socio-cultural and socio-economic issues

There was evidence of cultural beliefs about certain foods reported in studies conducted in Tanzania in 1997^44^ and in Kenya in 2009.^28^ Jilek-Aall et al.^44^ found that PWE were restricted from eating kambali (a local species of catfish), because it is slimy like foamy saliva, and chicken meat, especially the head and feet, because of the belief that they are used in traditional medicines. Although health consequences of these cultural beliefs about foods were not fully explored, one study conducted in rural Benin by Crepin et al.^45^ reported on health problems such as tooth decay and malnutrition. Similarly, Vaid et al.^46^ in rural Ethiopia found that malnutrition was because of socio-economic factors and poverty and that most CWE had stunted growth. However, the link between malnutrition and cultural beliefs about diet were not explored – showing a need for research in this area.

#### Strategies for addressing challenges of people with epilepsy and carers

There were some suggested strategies such as collaboration with mental health workers, patient advocacy and social marketing,^47^ a patient-centred approach^48^ and peer support groups to reduce stigma.^49^ However, Elafros et al.^49^ reiterate that the content of the peer support group must address the groups’ needs and that the use of local facilitators is crucial. Although these strategies were suggested, there was a lack of emphasis on health literacy – an aspect that is crucial for improving the understanding, accessing, processing and use of health information.^50^

## Ethical consideration

This literature review was part of a larger doctoral project that was approved by the four local research ethics committees (University of Cape Town-HREC 440/2011; Stellenbosch University-HS739/2011; PGWC-RP 163/2011 and City Health-ID 10272).

## Study limitations

Our review was not a systematic review – its aim was more modest: to gain insight into existing knowledge and to evidentially identify research gaps from reviewed studies on psychosocial, cultural and socio-economic related challenges faced by adult PWE and their carers. We acknowledge that we may have missed important evidence based on our inclusion and exclusion criteria and that our views, gender and backgrounds may have influenced our interpretation of the data.

## Conclusion

Our review provided an overview of key available literature on studies that examined the psychosocial difficulties experienced by adult PWE and their carers in Africa between 1994 and 2014. There is evidence that PWE and their carers experience different psychosocial challenges at different levels of the health system – and these have an enormous impact on their QOL. There is a need for research on psychosocial challenges of adults with epilepsy and their carers from different sectors of healthcare to inform the design of appropriate empowering interventions.
